# Supplementary material for: Hidden interactions in the intertidal rocky shore: variation in pedal mucus microbiota among marine grazers that feed on epilithic biofilm communities
Source: PeerJ. 2022 Sep 23;10:e13642. doi: 10.7717/peerj.13642 (PMC9512015; doi:10.7717/peerj.13642)

Supplement 1.

Table S1: Description, DNA extraction and 16S rRNA gene sequencing characteristics of the most abundant grazer´s microbiota and epilithic biofilm samples.

**ID**	**Treatment/ Microbiota**	**Foot Area (cm^2) or Rock Area (cm^2)**	**ng dDNA/uL**	**Number of QC reads**	**Number of ASVs**	**Number of reads after rarefaction with control**	**Number of ASVs after rarefaction with control**	**Number of reads after rarefaction without control**	**Number of ASVs after rarefaction without control**
C.granosus_47	*C. granosus*	8.05	44.6	14521	266	1869	215	10599	265
C.granosus_48	*C. granosus*	7.16	51	15543	192	1869	135	10599	190
C.granosus_49	*C. granosus*	5.11	46.8	16135	250	1869	195	10599	246
C.granosus_50	*C. granosus*	4.08	53.6	21317	255	1869	171	10599	244
E.peruviana_57	*E. peruviana*	0.09	37.4	29434	84	1869	51	10599	82
E.peruviana_58	*E. peruviana*	0.24	33.6	16898	125	1869	89	10599	122
E.peruviana_59	*E. peruviana*	0.22	12.4	74324	165	1869	72	10599	130
E.peruviana_60	*E. peruviana*	0.32	25.2	15918	94	1869	81	10599	93
F.crassa_38	*F. crassa*	31.10	45.4	21750	221	1869	169	10599	220
F.crassa_40	*F. crassa*	26.72	74.4	19390	212	1869	159	10599	204
F.crassa_53	*F. crassa*	15.37	52.2	10599	139	1869	120	10599	139
F.crassa_54	*F. crassa*	14.14	36.4	16151	158	1869	127	10599	154
F.crassa_55	*F. crassa*	27.90	59.6	15917	226	1869	169	10599	226
S.araucana_41	*S. Araucana*	7.05	59.2	54228	140	1869	86	10599	125
S.araucana_42	*S. Araucana*	0.43	33	29719	257	1869	141	10599	237
S.araucana_43	*S. Araucana*	0.06	28.4	34974	197	1869	118	10599	186
S.araucana_44	*S. Araucana*	0.23	14	75493	384	1869	191	10599	311
S.araucana_45	*S. Araucana*	0.37	45.2	23971	338	1869	221	10599	328
S.lessonii_62	*S. lessonii*	0.12	31	74566	145	1869	63	10599	128
S.lessonii_63	*S. lessonii*	0.11	55.2	28785	120	1869	75	10599	117
S.lessonii_64	*S. lessonii*	0.20	30	33081	138	1869	98	10599	134
S.lessonii_65	*S. lessonii*	0.17	65.4	11137	68	1869	61	10599	68
EpilithicBiofilm_1	Epilithic biofilm control	28.25	59	1869	118	1869	118	-	-
EpilithicBiofilm_5	Epilithic biofilm control	23.71	37.2	4449	101	1869	100	-	-
EpilithicBiofilm_27	Epilithic biofilm control	27.39	26.4	21089	400	1869	284	-	-
EpilithicBiofilm_29	Epilithic biofilm control	20.00	30.4	2236	111	1869	111	-	-
**TOTAL**	**-**	**-**	**-**	**683494**	**1934**	**48594**	**958**	**233178**	**1205**

Figure S1. First rarefaction curves with the epilithic biofilm control and the pedal mucus microbiota of *Chiton granosus, Echinolittorina peruviana, Fissurella crassa, Scurria araucana* and *Siphonaria lessonii.* Rarefaction level of 1,869 reads.

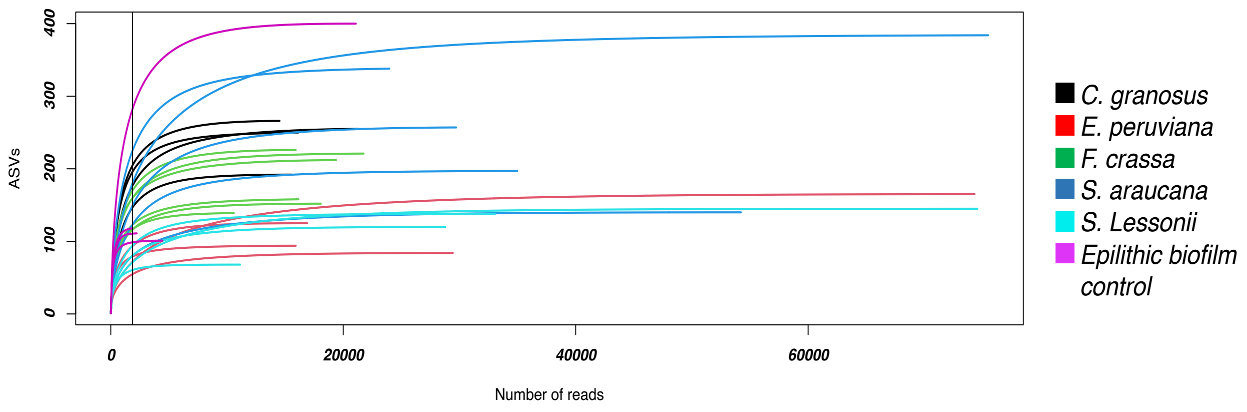

Figure S2. Second rarefaction curves with the pedal mucus microbiota of *Chiton granosus, Echinolittorina peruviana, Fissurella crassa, Scurria araucana* and *Siphonaria lessonii.* Rarefaction level of 10,599 reads.

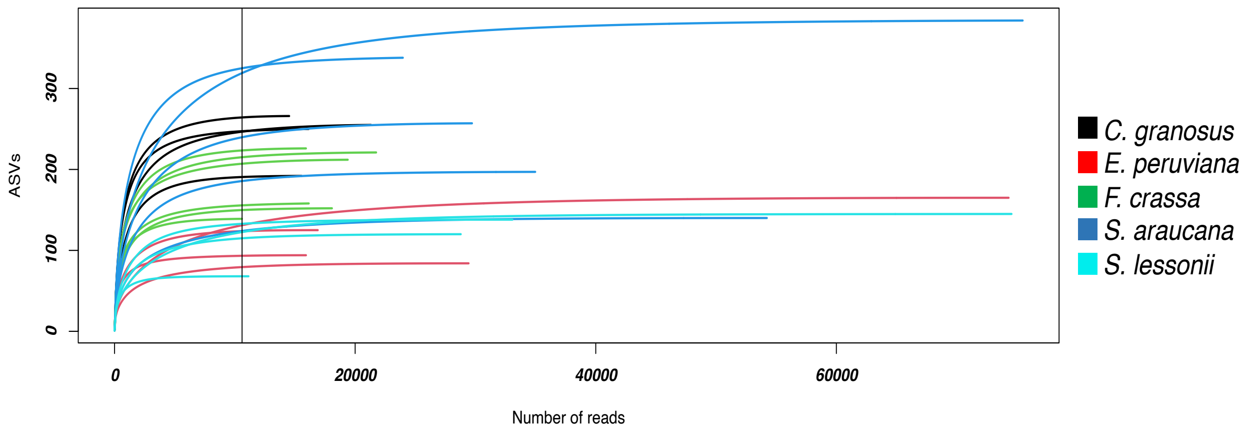

Supplement 2.

Figure S1. Pedal mucus stained with 0.01% acridine orange (right) and gram (left) of (A, B) *C. granosus,* (C, D) *E. peruviana*, (E, F) *F. crassa*, (G, H) *S. araucana,* and (I, J) *S. lessonii.* Photography by Clara Arboleda-Baena.

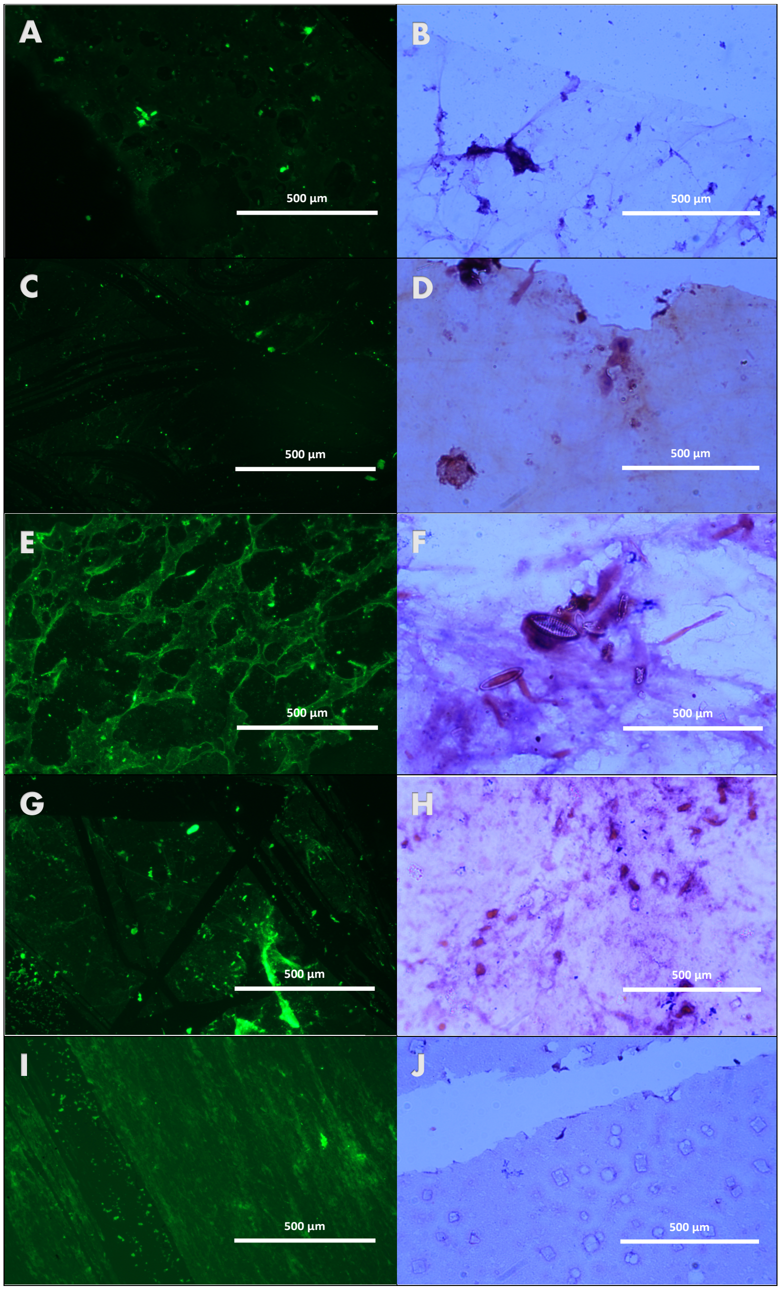

Supplement 3

Table S1: Taxa contribution percentage in the microbial community of pedal mucus microbiota of *Chiton granosus, Echinolittorina peruviana, Fissurella crassa, Scurria araucana* and *Siphonaria lessonii* and the Epilithic Biofilm Control. Red color percentage corresponds to the most abundant taxa of mayor microbial phyla, with a relative abundance of reads > 1% (Pedrós-Alió 2012). Rarefaction level <2000 reads.

TAXA	Epilithic Biofilm Control	Microbiota *C. granosus*	Microbiota *E. peruviana*	Microbiota *F. crassa*	Microbiota *S. araucana*	Microbiota *S. lessonii*
**Acidobacteria**	0.16	0.00	0.00	0.02	0.03	0.00
**Actinobacteria**	0.32	0.08	0.00	0.05	0.04	0.00
**Alphaproteobacteria**	**60.56**	**20.46**	**8.57**	**14.02**	**23.99**	**10.71**
**Bacteroidetes**	**12.86**	**66.52**	**8.09**	**67.29**	**49.88**	**5.09**
**Cyanobacteria**	0.17	0.08	0.03	0.08	0.14	0.00
**Dadabacteria**	0.24	0.00	0.00	0.00	0.00	0.00
**Deinococcus-Thermus**	0.00	0.00	0.00	0.00	0.00	0.03
**Deltaproteobacteria**	0.90	0.04	0.00	0.69	0.12	0.03
**Dependentiae**	0.59	0.00	0.00	0.00	0.00	0.00
**Epsilonbacteraeota**	0.04	0.17	**14.69**	0.96	0.58	**6.33**
**Firmicutes**	0.27	0.00	0.00	0.00	0.03	0.13
**Fusobacteria**	0.00	0.09	0.00	0.00	0.00	0.00
**Gammaproteobacteria**	**1.93**	**10.95**	**66.17**	**12.89**	**24.87**	**77.45**
**Other**	0.00	0.00	0.00	0.00	0.00	0.00
**Patescibacteria**	0.00	0.32	0.00	0.18	0.17	0.01
**Planctomycetes**	**19.74**	0.07	0.00	0.12	0.05	0.00
**Spirochaetes**	0.00	0.00	0.00	0.32	0.00	0.03
**Tenericutes**	0.00	0.00	0.00	0.03	0.00	0.03
**Thaumarchaeota**	0.05	0.00	0.00	0.00	0.00	0.00
**Unclassified**	0.06	0.00	0.00	0.00	0.00	0.00
**Verrucomicrobia**	**2.10**	**1.20**	**2.47**	**3.36**	0.08	0.16
**TOTAL**	**100**	**100**	**100**	**100**	**100**	**100**

Table S2: Total reads of the most abundant ASVs of the pedal mucus microbiota of *Chiton granosus, Echinolittorina peruviana, Fissurella crassa, Scurria araucana,* and *Siphonaria lessonii.* Rarefaction level of 10,599 reads.

Phylum	Class	Order	Family	*C.granosus*	*E.peruviana*	*F.crassa*	*S.araucana*	*S.lessonii*	TotalReads
**Bacteroidetes**	**Bacteroidia**	Chitinophagales	Saprospiraceae	149	11	13	31	6	209
		Cytophagales	Cyclobacteriaceae	46	12	69	15	5	146
		Flavobacteriales	Crocinitomicaceae	3152	0	827	233	0	4213
			Cryomorphaceae	339	0	60	292	7	699
			Flavobacteriaceae	3367	821	6291	4669	531	15678
**Epsilonbacteraeota**	**Campylobacteria**	Campylobacterales	Arcobacteraceae	14	1526	124	64	641	2369
**Proteobacteria**	**Alphaproteobacteria**	Rhodobacterales	Rhodobacteraceae	2060	848	1251	2435	1189	7782
	**Gammaproteobacteria**	Alteromonadales	Alteromonadaceae	307	781	17	240	1095	2439
			Colwelliaceae	368	3430	71	551	3900	8320
			Pseudoalteromonadaceae	29	40	73	416	133	691
			Shewanellaceae	17	8	47	30	9	112
		Cellvibrionales	Cellvibrionaceae	18	125	3	25	253	423
		Oceanospirillales	Marinomonadaceae	1	860	17	129	977	1984
			Nitrincolaceae	83	466	107	480	344	1479
			Saccharospirillaceae	35	1096	65	54	896	2146
		Vibrionales	Vibrionaceae	218	244	918	594	475	2447
**Verrucomicrobia**	**Verrucomicrobiae**	Verrucomicrobiales	Rubritaleaceae	130	245	276	11	19	681

Supplement 4.

Figure S1: Microbiota compositional similarity of the most abundant grazers of the intertidal rocky shore and the epilithic biofilm. Non-metric multidimensional scaling (NMDS) ordination plots based on Jaccard distances. The shapes denote the microbiota grazer species surrounded by an ellipse showing the 95% confidence interval. Stress = 0.101. Rarefaction level <2000 reads.

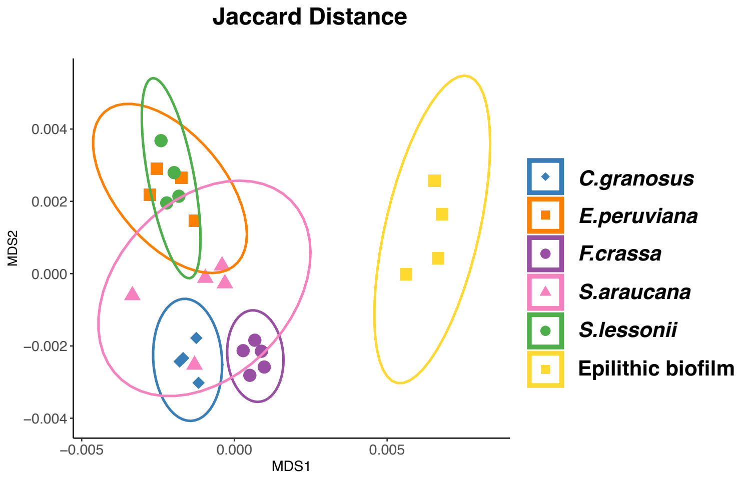

Table S1: Bray-Curtis distance. False Discovery Rate (FDR) corrected (Benjamini and Hochberg 1995) pairwise comparisons of the microbial communities of the pedal mucus microbiota of *Chiton granosus, Echinolittorina peruviana, Fissurella crassa, Scurria araucana,* and *Siphonaria lessonii* and the epilithic biofilms. Rarefaction level <2000 reads.

pairs	Df	Sums of Sqs	F. Model	R^2	p .value	p. adjusted sig
C.granosus vs *E.peruviana*	1	0.93	5.00	0.45	0.03	0.04
C.granosus vs *F.crassa*	1	1.04	6.94	0.50	0.01	0.02
C.granosus vs *S.araucana*	1	0.50	2.14	0.23	0.05	0.05
C.granosus vs *S.lessonii*	1	0.94	5.62	0.48	0.03	0.04
C.granosus vs Epilithic biofilm	1	1.42	8.04	0.57	0.03	0.04
E.peruviana vs *F.crassa*	1	1.34	9.67	0.58	0.01	0.02
E.peruviana vs *S.araucana*	1	0.76	3.45	0.33	0.01	0.02
E.peruviana vs *S.lessonii*	1	0.05	0.33	0.05	0.91	0.91
E.peruviana vs Epilithic biofilm	1	1.49	9.13	0.60	0.02	0.04
F.crassa vs *S.araucana*	1	1.00	5.41	0.40	0.01	0.02
F.crassa vs *S.lessonii*	1	1.37	11.20	0.62	0.01	0.02
F.crassa vs Epilithic biofilm	1	1.70	13.10	0.65	0.01	0.02
S.araucana vs *S.lessonii*	1	0.78	3.80	0.35	0.01	0.02
S.araucana vs Epilithic biofilm	1	1.44	6.79	0.49	0.01	0.02
S.lessonii vs Epilithic biofilm	1	1.55	10.73	0.64	0.03	0.04

Table S2: Jaccard distance. False Discovery Rate (FDR) corrected (Benjamini and Hochberg 1995) pairwise comparisons of the microbial communities of the pedal mucus microbiota of *Chiton granosus, Echinolittorina peruviana, Fissurella crassa, Scurria araucana,* and *Siphonaria lessonii* and the epilithic biofilms. Rarefaction level <2000 reads.

pairs	Df	Sums of Sqs	F. Model	R^2	p .value	p. adjusted sig
C.granosus vs E.peruviana	1	0.93	5.00	0.45	0.04	0.04
C.granosus vs F.crassa	1	1.04	6.94	0.50	0.01	0.02
C.granosus vs S.araucana	1	0.50	2.14	0.23	0.05	0.05
C.granosus vs S.lessonii	1	0.94	5.62	0.48	0.02	0.03
C.granosus vs Epilithic biofilm	1	1.42	8.04	0.57	0.03	0.04
E.peruviana vs F.crassa	1	1.34	9.67	0.58	0.00	0.02
E.peruviana vs S.araucana	1	0.76	3.45	0.33	0.01	0.02
E.peruviana vs S.lessonii	1	0.05	0.33	0.05	0.91	0.91
E.peruviana vs Epilithic biofilm	1	1.49	9.13	0.60	0.03	0.04
F.crassa vs S.araucana	1	1.00	5.41	0.40	0.01	0.02
F.crassa vs S.lessonii	1	1.37	11.20	0.62	0.02	0.03
F.crassa vs Epilithic biofilm	1	1.70	13.10	0.65	0.01	0.02
S.araucana vs S.lessonii	1	0.78	3.80	0.35	0.01	0.02
S.araucana vs Epilithic biofilm	1	1.44	6.79	0.49	0.01	0.02
S.lessonii vs Epilithic biofilm	1	1.55	10.73	0.64	0.02	0.03

Figure S2: Microbiota compositional similarity of the most abundant grazers of the intertidal rocky shore. Non-metric multidimensional scaling (NMDS) ordination plots based on (A) Bray-Curtis and (B) Jaccard distances. The shapes denote the microbiota grazer species surrounded by an ellipse showing the 95% confidence interval. (A) Stress = 0.112, (B) stress = 0.112. Rarefaction level of 10,599 reads.

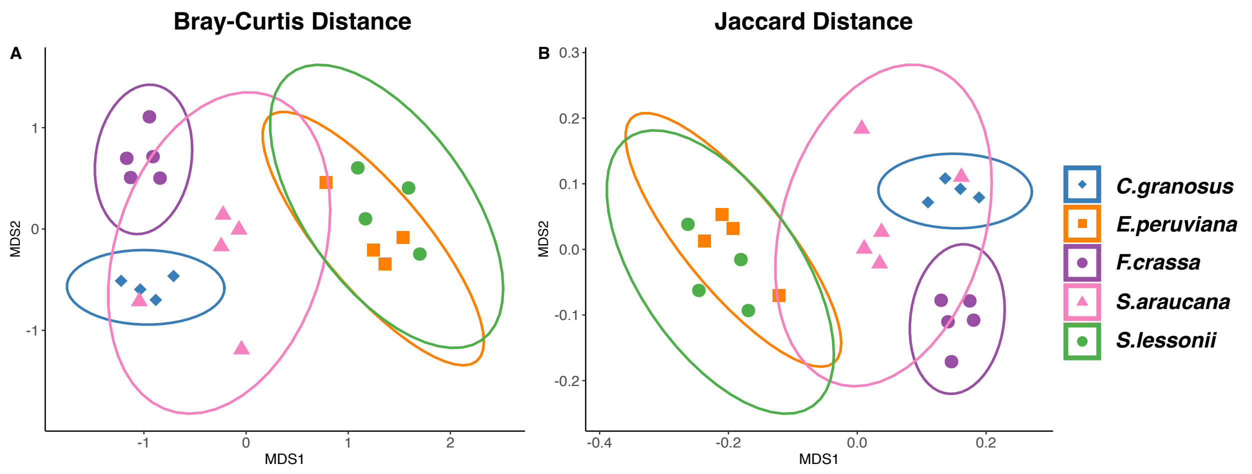

Table S3: Bray-Curtis distance. Permanova p > 0.001. False Discovery Rate (FDR) corrected (Benjamini and Hochberg 1995) pairwise comparisons of the microbial communities of the pedal mucus microbiota of *Chiton granosus, Echinolittorina peruviana, Fissurella crassa, Scurria araucana,* and *Siphonaria lessonii.* Rarefaction level of 10,599 reads.

pairs	Df	SumsOfSqs	F.Model	R2	p.value	p.adjusted	sig
C.granosus vs E.peruviana	1	0.95	5.38	0.47	0.03	0.04	.
C.granosus vs F.crassa	1	1.04	7.41	0.51	0.01	0.02	.
C.granosus vs S.araucana	1	0.51	2.23	0.24	0.04	0.04	.
C.granosus vs S.lessonii	1	0.93	5.88	0.50	0.03	0.04	.
E.peruviana vs F.crassa	1	1.36	10.22	0.59	0.01	0.02	.
E.peruviana vs S.araucana	1	0.78	3.55	0.34	0.01	0.02	.
E.peruviana vs S.lessonii	1	0.05	0.34	0.05	0.93	0.93	
F.crassa vs S.araucana	1	1.01	5.53	0.41	0.01	0.02	.
F.crassa vs S.lessonii	1	1.38	11.74	0.63	0.01	0.02	.
S.araucana vs S.lessonii	1	0.77	3.76	0.35	0.01	0.02	.

Table S4: Jaccard distance. Permanova p > 0.001. False Discovery Rate (FDR) corrected (Benjamini and Hochberg 1995) pairwise comparisons of the microbial communities of the pedal mucus microbiota of *Chiton granosus, Echinolittorina peruviana, Fissurella crassa, Scurria araucana,* and *Siphonaria lessonii.* Rarefaction level of 10,599 reads.

pairs	Df	SumsOfSqs	F.Model	R2	p.value	p.adjusted	sig
C.granosus vs E.peruviana	1	0.95	5.38	0.47	0.03	0.03	.
C.granosus vs F.crassa	1	1.04	7.41	0.51	0.01	0.02	.
C.granosus vs S.araucana	1	0.51	2.23	0.24	0.02	0.03	.
C.granosus vs S.lessonii	1	0.93	5.88	0.50	0.02	0.03	.
E.peruviana vs F.crassa	1	1.36	10.22	0.59	0.01	0.02	.
E.peruviana vs S.araucana	1	0.78	3.55	0.34	0.01	0.02	.
E.peruviana vs S.lessonii	1	0.05	0.34	0.05	0.91	0.91	
F.crassa vs S.araucana	1	1.01	5.53	0.41	0.01	0.02	.
F.crassa vs S.lessonii	1	1.38	11.74	0.63	0.01	0.02	.
S.araucana vs S.lessonii	1	0.77	3.76	0.35	0.01	0.02	.

Figure S3. PERMDISP analyses for a dataset of rarefaction level <2000 reads. The figure showed the distance to the centroid of *C.granosus, E.peruviana, F. crassa, S.araucana,* and *S. lessonii* and the epilithic biofilms group.

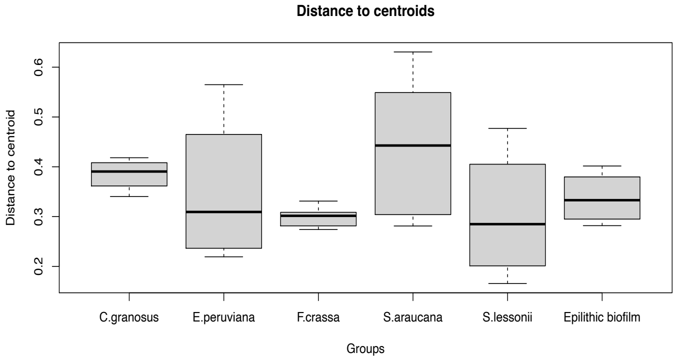

Analysis of Variance Table (Response: Distances)

	Df	Sum Sq.	Mean.Sq	F value	Pr(>F)
Groups	5	0.068535	0.013707	1.176	0.3555
Residuals	20	0.233119	0.011656		

Figure S4. PERMDISP analyses for a dataset of rarefaction level 10,599 reads. The figure showed the distance to the centroid of *C.granosus, E.peruviana, F. crassa, S.araucana,* and *S. lessonii* group.

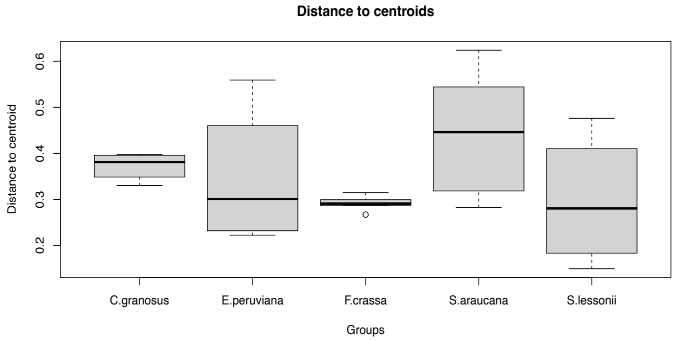

Analysis of Variance Table (Response: Distances)

	Df	Sum Sq	Mean Sq	F value	Pr(>F)
Groups	4	0.073608	0.018402	1.4046.	0.2747
Residuals	17	0.222730	0.013102		

Supplement 5

Figure S1. Mean **A**) richness and **B**) Shannon index diversity of microbial community (Bacteria and Archaea) of the pedal mucus microbiota of *C. granosus, F. crassa, S. araucana, E, peruviana, S. lessonii* and the epilithic biofilm (mean + SE). Different letters above bars indicate significant differences (among treatments from Tukey test at the experiment-wise error rate = 0.05). Rarefaction level of 1,869 reads.

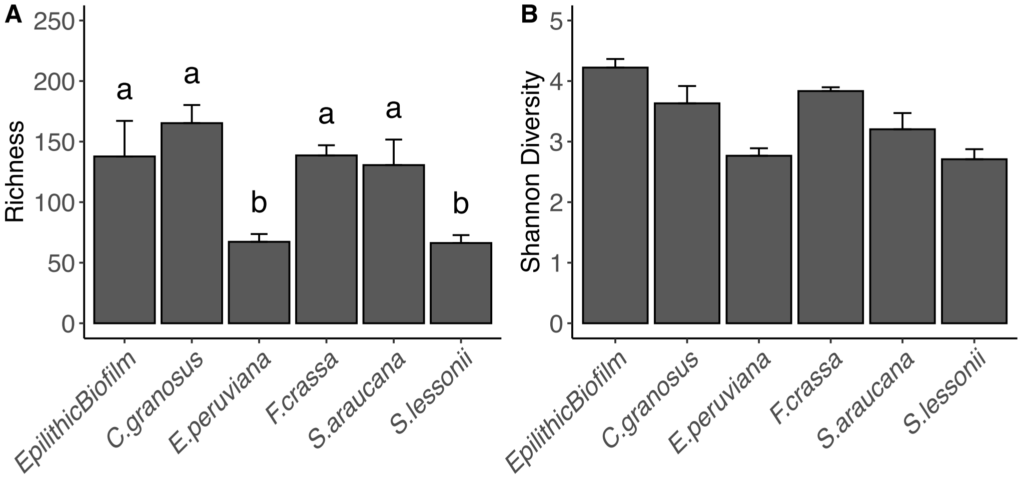

Figure S2. Mean **A**) richness and **B**) Shannon index diversity of the microbial community (Bacteria and Archaea) of the pedal mucus microbiota of *C. granosus, F. crassa, S. araucana, E, peruviana, S. lessonii* (mean + SE). Different letters above bars indicate significant differences (among treatments from Tukey test at the experiment-wise error rate = 0.05). Rarefaction level of 10,599 reads.

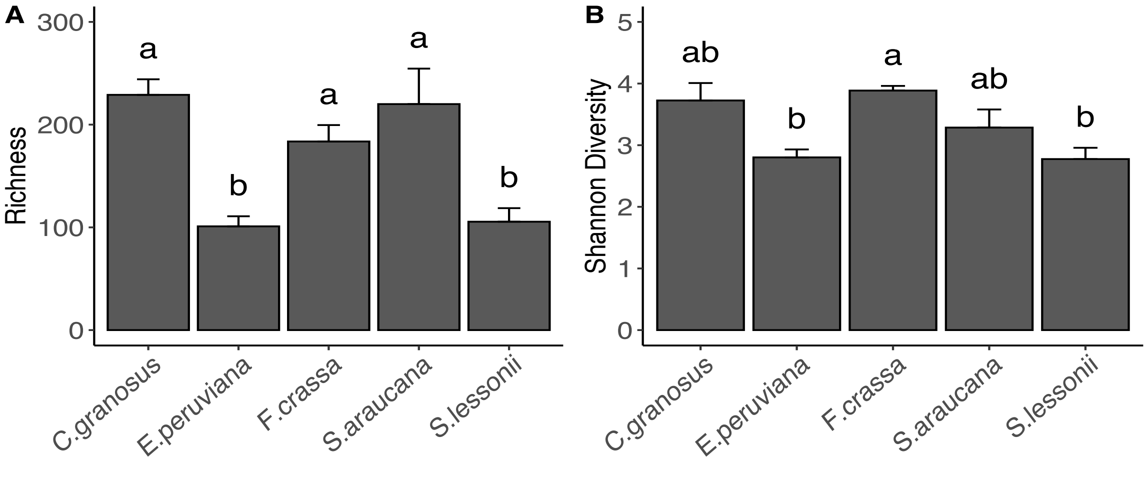

Supplement 6.

Table S1: Tukey post hoc test multiple comparisons of richness of the microbial communities of the pedal mucus microbiota and the epilithic biofilms. 95% family-wise confidence level. Rarefaction level of 1,869 reads.

**Comparisons**	**diff**	**lwr**	**upr**	**p adj**
E.peruviana-C.granosus	-0.391	-0.648	-0.134	0.001
F.crassa-C.granosus	-0.074	-0.318	0.170	0.927
S.araucana-C.granosus	-0.120	-0.364	0.123	0.636
S.lessonii-C.granosus	-0.397	-0.654	-0.140	0.001
EpilithicBiofilm-*C.granosus*	-0.098	-0.355	0.159	0.831
F.crassa-E.peruviana	0.317	0.074	0.561	0.006
S.araucana-E.peruviana	0.271	0.027	0.514	0.024
S.lessonii-E.peruviana	-0.006	-0.263	0.251	1.000
EpilithicBiofilm-*E.peruviana*	0.293	0.036	0.550	0.020
S.araucana-F.crassa	-0.046	-0.276	0.183	0.987
S.lessonii-F.crassa	-0.323	-0.567	-0.080	0.005
EpilithicBiofilm-*F.crassa*	-0.024	-0.268	0.219	1.000
S.lessonii-S.araucana	-0.277	-0.521	-0.033	0.020
EpilithicBiofilm-*S.araucana*	0.022	-0.221	0.266	1.000
EpilithicBiofilm-*S.lessonii*	0.299	0.042	0.556	0.017

Table S2: Tukey post hoc test multiple comparisons of Shannon index of the microbial communities of the pedal mucus microbiota and the epilithic biofilms. 95% family-wise confidence level. Rarefaction level of 1,869 reads.

**Comparisons**	**diff**	**lwr**	**upr**	**p adj**
E.peruviana-C.granosus	-0.115	-0.236	0.006	0.069
EpilithicBiofilm-*C.granosus*	0.069	-0.052	0.190	0.492
F.crassa-C.granosus	0.028	-0.087	0.143	0.971
S.araucana-C.granosus	-0.056	-0.171	0.059	0.649
S.lessonii-C.granosus	-0.126	-0.247	-0.004	0.040
EpilithicBiofilm-*E.peruviana*	0.184	0.063	0.306	0.001
F.crassa-E.peruviana	0.143	0.028	0.258	0.010
S.araucana-E.peruviana	0.059	-0.056	0.174	0.599
S.lessonii-E.peruviana	-0.010	-0.132	0.111	1.000
F.crassa-EpilithicBiofilm	-0.041	-0.156	0.074	0.863
S.araucana-EpilithicBiofilm	-0.125	-0.240	-0.010	0.028
S.lessonii-EpilithicBiofilm	-0.195	-0.316	-0.073	0.001
S.araucana-F.crassa	-0.084	-0.192	0.025	0.193
S.lessonii-F.crassa	-0.153	-0.268	-0.038	0.005
S.lessonii-S.araucana	-0.070	-0.185	0.045	0.430

Table S3: Tukey post hoc test multiple comparisons of richness of the microbial communities of the pedal mucus microbiota. 95% family-wise confidence level. Dataset with a rarefaction level of 10,599 reads.

Comparisons	diff	lwr	upr	p adj
**E.peruviana-C.granosus**	-0.359	-0.607	-0.111	0.003
**F.crassa-C.granosus**	-0.100	-0.335	0.136	0.700
**S.araucana-C.granosus**	-0.039	-0.275	0.197	0.986
**S.lessonii-C.granosus**	-0.346	-0.594	-0.097	0.004
**F.crassa-E.peruviana**	0.259	0.023	0.495	0.027
**S.araucana-E.peruviana**	0.320	0.084	0.556	0.005
**S.lessonii-E.peruviana**	0.013	-0.235	0.262	1.000
**S.araucana-F.crassa**	0.061	-0.161	0.283	0.916
**S.lessonii-F.crassa**	-0.246	-0.481	-0.010	0.039
**S.lessonii-S.araucana**	-0.307	-0.542	-0.071	0.008

Table S4: Tukey post hoc test multiple comparisons of Shannon index of the microbial communities of the pedal mucus microbiota. 95% family-wise confidence level. Dataset with a rarefaction level of 10,599 reads.

Comparisons	diff	lwr	upr	p adj
**E.peruviana-C.granosus**	-0.121	-0.250	0.008	0.073
**F.crassa-C.granosus**	0.022	-0.101	0.145	0.981
**S.araucana-C.granosus**	-0.057	-0.180	0.065	0.622
**S.lessonii-C.granosus**	-0.127	-0.256	0.002	0.056
**F.crassa-E.peruviana**	0.143	0.020	0.266	0.018
**S.araucana-E.peruviana**	0.064	-0.059	0.186	0.530
**S.lessonii-E.peruviana**	-0.006	-0.135	0.123	1.000
**S.araucana-F.crassa**	-0.079	-0.195	0.036	0.269
**S.lessonii-F.crassa**	-0.149	-0.272	-0.026	0.013
**S.lessonii-S.araucana**	-0.070	-0.192	0.053	0.446

Supplement 7

Table S1: ASVs habitat specialist to a specific pedal mucus microbiota of *Chiton granosus, Echinolittorina peruviana, Fissurella crassa, Scurria araucana* and *Siphonaria lessonii* with the associated probability (p). Number of reads and ASVs Taxa classification is also presented. Dataset with a rarefaction level of 10,599 reads.

ASV	Number of reads	pVal	Microbiota	Phylum	Class	Order	Family	Genus
**ASV_12**	8759	0.017	*E.peruviana*	Epsilonbacteraeota	Campylobacteria	Campylobacterales	Arcobacteraceae	Arcobacter
**ASV_43**	8468	0.009	*C.granosus*	Bacteroidetes	Bacteroidia	Flavobacteriales	Crocinitomicaceae	Crocinitomix
**ASV_16**	6440	0.038	*E.peruviana*	Proteobacteria	Gammaproteobacteria	Alteromonadales	Colwelliaceae	Colwellia
**ASV_22**	6184	0.024	*S.araucana*	Proteobacteria	Alphaproteobacteria	Rhodobacterales	Rhodobacteraceae	Pseudophaeobacter
**ASV_56**	5505	0.001	*F.crassa*	Bacteroidetes	Bacteroidia	Flavobacteriales	Flavobacteriaceae	Tenacibaculum
**ASV_26**	4781	0.004	*S.araucana*	Bacteroidetes	Bacteroidia	Flavobacteriales	Flavobacteriaceae	Polaribacter
**ASV_98**	3722	0.009	*F.crassa*	Bacteroidetes	Bacteroidia	Flavobacteriales	Flavobacteriaceae	NA
**ASV_134**	3443	0.001	*F.crassa*	Bacteroidetes	Bacteroidia	Flavobacteriales	Flavobacteriaceae	Polaribacter_4
**ASV_51**	3269	0.024	*S.araucana*	Proteobacteria	Alphaproteobacteria	Rhodobacterales	Rhodobacteraceae	Roseobacter_clade_NAC11-7_lineage
**ASV_139**	3224	0.001	*F.crassa*	Bacteroidetes	Bacteroidia	Flavobacteriales	Flavobacteriaceae	Tenacibaculum
**ASV_23**	2663	0.023	*E.peruviana*	Proteobacteria	Gammaproteobacteria	Oceanospirillales	Nitrincolaceae	Neptuniibacter
**ASV_232**	2181	0.001	*F.crassa*	Bacteroidetes	Bacteroidia	Flavobacteriales	Flavobacteriaceae	NA
**ASV_163**	2153	0.003	*C.granosus*	Bacteroidetes	Bacteroidia	Flavobacteriales	Crocinitomicaceae	Crocinitomix
**ASV_208**	2040	0.006	*C.granosus*	Bacteroidetes	Bacteroidia	Flavobacteriales	Flavobacteriaceae	Olleya
**ASV_166**	1942	0.026	*C.granosus*	Bacteroidetes	Bacteroidia	Flavobacteriales	Flavobacteriaceae	Olleya
**ASV_82**	1926	0.001	*S.araucana*	Bacteroidetes	Bacteroidia	Flavobacteriales	Flavobacteriaceae	NA
**ASV_234**	1676	0.04	*C.granosus*	Bacteroidetes	Bacteroidia	Flavobacteriales	Crocinitomicaceae	Crocinitomix
**ASV_83**	1647	0.03	*E.peruviana*	Proteobacteria	Gammaproteobacteria	Alteromonadales	Colwelliaceae	Colwellia
**ASV_138**	1484	0.001	*F.crassa*	Bacteroidetes	Bacteroidia	Flavobacteriales	Flavobacteriaceae	Polaribacter_4
**ASV_312**	1478	0.002	*C.granosus*	Bacteroidetes	Bacteroidia	Flavobacteriales	Flavobacteriaceae	Olleya
**ASV_274**	1464	0.001	*F.crassa*	Bacteroidetes	Bacteroidia	Flavobacteriales	Flavobacteriaceae	Nonlabens
**ASV_150**	1435	0.001	*S.araucana*	Bacteroidetes	Bacteroidia	Flavobacteriales	Flavobacteriaceae	Pseudofulvibacter
**ASV_316**	1200	0.001	*F.crassa*	Bacteroidetes	Bacteroidia	Flavobacteriales	Flavobacteriaceae	Lacinutrix
**ASV_131**	1170	0.001	*S.araucana*	Proteobacteria	Gammaproteobacteria	Oceanospirillales	Nitrincolaceae	Marinobacterium
**ASV_302**	959	0.001	*F.crassa*	Proteobacteria	Alphaproteobacteria	Rhodobacterales	Rhodobacteraceae	Roseobacter
**ASV_264**	951	0.012	*F.crassa*	Proteobacteria	Gammaproteobacteria	Oceanospirillales	Nitrincolaceae	Amphritea
**ASV_256**	870	0.001	*S.lessonii*	Proteobacteria	Gammaproteobacteria	Alteromonadales	Alteromonadaceae	Paraglaciecola
**ASV_586**	857	0.001	*F.crassa*	Bacteroidetes	Bacteroidia	Flavobacteriales	Flavobacteriaceae	Polaribacter_4
**ASV_509**	829	0.008	*C.granosus*	Bacteroidetes	Bacteroidia	Flavobacteriales	Crocinitomicaceae	Salinirepens
**ASV_57**	817	0.022	*S.lessonii*	Proteobacteria	Gammaproteobacteria	Alteromonadales	Alteromonadaceae	Alteromonas
**ASV_307**	807	0.001	*S.araucana*	Bacteroidetes	Bacteroidia	Flavobacteriales	Flavobacteriaceae	Tenacibaculum
**ASV_446**	807	0.002	*C.granosus*	Bacteroidetes	Bacteroidia	Flavobacteriales	Cryomorphaceae	NS10_marine_group
**ASV_486**	802	0.003	*F.crassa*	Verrucomicrobia	Verrucomicrobiae	Verrucomicrobiales	Rubritaleaceae	Rubritalea
**ASV_504**	796	0.001	*F.crassa*	Bacteroidetes	Bacteroidia	Flavobacteriales	Flavobacteriaceae	Tenacibaculum
**ASV_32**	741	0.003	*S.lessonii*	Proteobacteria	Gammaproteobacteria	Cellvibrionales	Cellvibrionaceae	Aestuariicella
**ASV_27**	730	0.013	*F.crassa*	Bacteroidetes	Bacteroidia	Flavobacteriales	Flavobacteriaceae	Polaribacter

**ASV_473**	729	0.014	*F.crassa*	Bacteroidetes	Bacteroidia	Flavobacteriales	Flavobacteriaceae	Lacinutrix
**ASV_546**	729	0.001	*F.crassa*	Bacteroidetes	Bacteroidia	Flavobacteriales	Flavobacteriaceae	NA
**ASV_240**	657	0.011	*E.peruviana*	Epsilonbacteraeota	Campylobacteria	Campylobacterales	Arcobacteraceae	Arcobacter
**ASV_581**	554	0.001	*F.crassa*	Bacteroidetes	Bacteroidia	Flavobacteriales	Flavobacteriaceae	Tenacibaculum
**ASV_640**	554	0.001	*F.crassa*	Bacteroidetes	Bacteroidia	Flavobacteriales	Flavobacteriaceae	NA
**ASV_306**	532	0.002	*S.araucana*	Proteobacteria	Gammaproteobacteria	Oceanospirillales	Nitrincolaceae	Marinobacterium
**ASV_734**	492	0.001	*F.crassa*	Verrucomicrobia	Verrucomicrobiae	Verrucomicrobiales	Rubritaleaceae	Rubritalea
**ASV_728**	481	0.005	*C.granosus*	Bacteroidetes	Bacteroidia	Flavobacteriales	Flavobacteriaceae	Flavirhabdus
**ASV_719**	444	0.002	*F.crassa*	Bacteroidetes	Bacteroidia	Flavobacteriales	Crocinitomicaceae	NA
**ASV_30**	442	0.001	*F.crassa*	Proteobacteria	Alphaproteobacteria	Rhodobacterales	Rhodobacteraceae	Loktanella
**ASV_810**	436	0.006	*F.crassa*	Bacteroidetes	Bacteroidia	Flavobacteriales	Flavobacteriaceae	NA
**ASV_717**	417	0.008	*C.granosus*	Bacteroidetes	Bacteroidia	Flavobacteriales	Flavobacteriaceae	Aurantivirga
**ASV_782**	414	0.001	*F.crassa*	Bacteroidetes	Bacteroidia	Flavobacteriales	Flavobacteriaceae	Polaribacter_3
**ASV_160**	412	0.005	*E.peruviana*	Proteobacteria	Gammaproteobacteria	Oceanospirillales	Nitrincolaceae	Neptuniibacter
**ASV_624**	410	0.01	*F.crassa*	Proteobacteria	Alphaproteobacteria	Rhodobacterales	Rhodobacteraceae	NA
**ASV_463**	400	0.035	*F.crassa*	Bacteroidetes	Bacteroidia	Flavobacteriales	Flavobacteriaceae	Kordia
**ASV_712**	400	0.006	*C.granosus*	Bacteroidetes	Bacteroidia	Flavobacteriales	Crocinitomicaceae	Crocinitomix
**ASV_803**	385	0.001	*F.crassa*	Bacteroidetes	Bacteroidia	Flavobacteriales	Flavobacteriaceae	NA
**ASV_972**	373	0.031	*F.crassa*	Bacteroidetes	Bacteroidia	Flavobacteriales	Flavobacteriaceae	Tenacibaculum
**ASV_986**	354	0.002	*F.crassa*	Bacteroidetes	Bacteroidia	Flavobacteriales	Flavobacteriaceae	NA
**ASV_899**	339	0.003	*C.granosus*	Bacteroidetes	Bacteroidia	Flavobacteriales	Flavobacteriaceae	NA
**ASV_815**	318	0.001	*F.crassa*	Bacteroidetes	Bacteroidia	Flavobacteriales	Flavobacteriaceae	Dokdonia
**ASV_558**	316	0.011	*S.lessonii*	Proteobacteria	Alphaproteobacteria	Rhodobacterales	Rhodobacteraceae	Loktanella
**ASV_540**	315	0.001	*S.araucana*	Bacteroidetes	Bacteroidia	Flavobacteriales	Flavobacteriaceae	Tenacibaculum
**ASV_537**	312	0.006	*C.granosus*	Bacteroidetes	Bacteroidia	Flavobacteriales	Flavobacteriaceae	Polaribacter_4
**ASV_715**	300	0.004	*S.araucana*	Proteobacteria	Alphaproteobacteria	Rhodobacterales	Rhodobacteraceae	NA
**ASV_753**	298	0.001	*F.crassa*	Bacteroidetes	Bacteroidia	Flavobacteriales	Crocinitomicaceae	Crocinitomix
**ASV_771**	290	0.001	*F.crassa*	Bacteroidetes	Bacteroidia	Cytophagales	Cyclobacteriaceae	NA
**ASV_754**	289	0.003	*C.granosus*	Bacteroidetes	Bacteroidia	Flavobacteriales	Flavobacteriaceae	Tenacibaculum
**ASV_992**	289	0.002	*C.granosus*	Bacteroidetes	Bacteroidia	Flavobacteriales	Flavobacteriaceae	NA
**ASV_366**	287	0.026	*S.araucana*	Bacteroidetes	Bacteroidia	Flavobacteriales	Flavobacteriaceae	Tenacibaculum
**ASV_668**	264	0.001	*F.crassa*	Bacteroidetes	Bacteroidia	Flavobacteriales	Flavobacteriaceae	Pseudofulvibacter
**ASV_354**	257	0.001	*F.crassa*	Proteobacteria	Gammaproteobacteria	Alteromonadales	Pseudoalteromonadaceae	Pseudoalteromonas
**ASV_1157**	249	0.011	*F.crassa*	Bacteroidetes	Bacteroidia	Flavobacteriales	Flavobacteriaceae	Tenacibaculum
**ASV_854**	248	0.041	*C.granosus*	Proteobacteria	Alphaproteobacteria	Rhodobacterales	Rhodobacteraceae	Roseobacter
**ASV_1062**	231	0.001	*F.crassa*	Bacteroidetes	Bacteroidia	Flavobacteriales	Flavobacteriaceae	NA
**ASV_858**	230	0.001	*F.crassa*	Bacteroidetes	Bacteroidia	Flavobacteriales	Flavobacteriaceae	Algibacter
**ASV_1038**	230	0.002	*F.crassa*	Proteobacteria	Deltaproteobacteria	Bdellovibrionales	Bacteriovoracaceae	Peredibacter
**ASV_1224**	227	0.009	*C.granosus*	Bacteroidetes	Bacteroidia	Flavobacteriales	Flavobacteriaceae	Tenacibaculum
**ASV_1193**	223	0.007	*C.granosus*	Bacteroidetes	Bacteroidia	Flavobacteriales	Flavobacteriaceae	NA
**ASV_1029**	221	0.021	*C.granosus*	Bacteroidetes	Bacteroidia	Flavobacteriales	Flavobacteriaceae	Pricia
**ASV_1133**	217	0.001	*C.granosus*	Proteobacteria	Alphaproteobacteria	Rhodobacterales	Rhodobacteraceae	Thalassobius
**ASV_1050**	214	0.04	*C.granosus*	Proteobacteria	Gammaproteobacteria	Alteromonadales	Alteromonadaceae	Paraglaciecola
**ASV_1274**	204	0.001	*F.crassa*	Bacteroidetes	Bacteroidia	Flavobacteriales	Flavobacteriaceae	NA
**ASV_1429**	199	0.001	*F.crassa*	Bacteroidetes	Bacteroidia	Flavobacteriales	Flavobacteriaceae	Tenacibaculum
**ASV_1278**	195	0.008	*F.crassa*	Proteobacteria	Alphaproteobacteria	Rhodobacterales	Rhodobacteraceae	Pseudophaeobacter
**ASV_1364**	194	0.005	*C.granosus*	Bacteroidetes	Bacteroidia	Flavobacteriales	Flavobacteriaceae	NA
**ASV_595**	180	0.004	*C.granosus*	Bacteroidetes	Bacteroidia	Flavobacteriales	Flavobacteriaceae	Tenacibaculum
**ASV_1174**	177	0.005	*F.crassa*	Proteobacteria	Gammaproteobacteria	Vibrionales	Vibrionaceae	Vibrio

**ASV_1489**	172	0.004	*C.granosus*	Verrucomicrobia	Verrucomicrobiae	Verrucomicrobiales	Rubritaleaceae	Rubritalea
**ASV_864**	169	0.001	*F.crassa*	Bacteroidetes	Bacteroidia	Flavobacteriales	Flavobacteriaceae	Aquibacter
**ASV_1459**	167	0.001	*F.crassa*	Spirochaetes	Spirochaetia	Spirochaetales	Spirochaetaceae	NA
**ASV_1639**	165	0.001	*C.granosus*	Bacteroidetes	Bacteroidia	Flavobacteriales	Flavobacteriaceae	Nonlabens
**ASV_1551**	162	0.001	*F.crassa*	Proteobacteria	Gammaproteobacteria	Cardiobacteriales	Cardiobacteriaceae	NA
**ASV_1523**	161	0.001	*C.granosus*	Bacteroidetes	Bacteroidia	Cytophagales	Cyclobacteriaceae	Persicobacter
**ASV_825**	152	0.001	*E.peruviana*	Verrucomicrobia	Verrucomicrobiae	Verrucomicrobiales	Rubritaleaceae	Rubritalea
**ASV_1536**	152	0.001	*F.crassa*	Proteobacteria	Deltaproteobacteria	Bdellovibrionales	Bacteriovoracaceae	NA
**ASV_1730**	152	0.003	*F.crassa*	Bacteroidetes	Bacteroidia	Flavobacteriales	Flavobacteriaceae	Pricia
**ASV_987**	150	0.001	*S.araucana*	Bacteroidetes	Bacteroidia	Flavobacteriales	Flavobacteriaceae	Aurantivirga
**ASV_1654**	149	0.001	*C.granosus*	Bacteroidetes	Bacteroidia	Chitinophagales	Saprospiraceae	NA
**ASV_96**	146	0.015	*C.granosus*	Proteobacteria	Alphaproteobacteria	Rhodobacterales	Rhodobacteraceae	Sulfitobacter
**ASV_145**	146	0.001	*F.crassa*	Proteobacteria	Alphaproteobacteria	Rhodobacterales	Rhodobacteraceae	Planktotalea
**ASV_1335**	144	0.043	*C.granosus*	Patescibacteria	Gracilibacteria	JGI_0000069-P22	NA	NA
**ASV_1558**	140	0.011	*C.granosus*	Bacteroidetes	Bacteroidia	Flavobacteriales	Flavobacteriaceae	Olleya
**ASV_964**	138	0.035	*E.peruviana*	Bacteroidetes	Bacteroidia	Flavobacteriales	Flavobacteriaceae	NA
**ASV_1377**	135	0.002	*F.crassa*	Proteobacteria	Gammaproteobacteria	Alteromonadales	Shewanellaceae	Shewanella
**ASV_700**	134	0.002	*S.araucana*	Proteobacteria	Gammaproteobacteria	Oceanospirillales	Nitrincolaceae	Neptuniibacter
**ASV_1658**	134	0.002	*C.granosus*	Bacteroidetes	Bacteroidia	Flavobacteriales	Flavobacteriaceae	Dokdonia
**ASV_481**	130	0.003	*F.crassa*	Bacteroidetes	Bacteroidia	Flavobacteriales	Flavobacteriaceae	Dokdonia
**ASV_1848**	128	0.003	*C.granosus*	Bacteroidetes	Bacteroidia	Flavobacteriales	Flavobacteriaceae	Winogradskyella
**ASV_1417**	127	0.009	*E.peruviana*	Bacteroidetes	Bacteroidia	Flavobacteriales	Flavobacteriaceae	Polaribacter_4
**ASV_879**	125	0.033	*S.araucana*	Bacteroidetes	Bacteroidia	Flavobacteriales	Flavobacteriaceae	NA
**ASV_1871**	124	0.01	*C.granosus*	Bacteroidetes	Bacteroidia	Flavobacteriales	Flavobacteriaceae	Dokdonia
**ASV_1043**	122	0.041	*S.araucana*	Bacteroidetes	Bacteroidia	Flavobacteriales	Flavobacteriaceae	Nonlabens
**ASV_1434**	117	0.004	*C.granosus*	Proteobacteria	Alphaproteobacteria	Rhizobiales	Phyllobacteriaceae	Ahrensia
**ASV_1659**	117	0.001	*C.granosus*	Bacteroidetes	Bacteroidia	Flavobacteriales	Flavobacteriaceae	Maribacter
**ASV_1118**	116	0.005	*S.araucana*	Bacteroidetes	Bacteroidia	Flavobacteriales	Flavobacteriaceae	Pseudofulvibacter
**ASV_934**	115	0.027	*S.araucana*	Proteobacteria	Gammaproteobacteria	Oceanospirillales	Nitrincolaceae	Profundimonas
**ASV_1918**	115	0.002	*C.granosus*	Bacteroidetes	Bacteroidia	Flavobacteriales	Flavobacteriaceae	Tamlana
**ASV_2076**	115	0.001	*F.crassa*	Verrucomicrobia	Verrucomicrobiae	Verrucomicrobiales	Rubritaleaceae	Persicirhabdus
**ASV_1921**	114	0.003	*C.granosus*	Bacteroidetes	Bacteroidia	Flavobacteriales	Flavobacteriaceae	Winogradskyella
**ASV_1640**	111	0.028	*C.granosus*	Proteobacteria	Alphaproteobacteria	Rhodobacterales	Rhodobacteraceae	Aliiroseovarius
**ASV_1990**	108	0.005	*C.granosus*	Bacteroidetes	Bacteroidia	Flavobacteriales	Flavobacteriaceae	Dokdonia
**ASV_2275**	108	0.018	*F.crassa*	Proteobacteria	Gammaproteobacteria	Cardiobacteriales	Cardiobacteriaceae	NA
**ASV_1058**	104	0.03	*S.araucana*	Bacteroidetes	Bacteroidia	Flavobacteriales	Flavobacteriaceae	Aquimarina
**ASV_1119**	104	0.012	*S.lessonii*	Proteobacteria	Gammaproteobacteria	Oceanospirillales	Marinomonadaceae	Marinomonas
**ASV_175**	101	0.003	*C.granosus*	Proteobacteria	Alphaproteobacteria	Rhodobacterales	Rhodobacteraceae	Oceanibulbus

Supplement 8.

Table S1: Tukey post hoc test multiple comparisons of carbohydrates content of the mollusk pedal mucus. 95% family-wise confidence level.

Comparisons	diff	lwr	upr	p adj
E. peruviana-C. granosus	2.221739	-19.699616	24.143094	0.9979968
F. crassa-C. granosus	29.765217	7.843862	51.686573	0.0048901
S. araucana-C. granosus	-9.078261	-30.999616	12.843094	0.7293341
S. lessonii-C. granosus	-1.373913	-23.295268	20.547442	0.9996977
F. crassa-E. peruviana	27.543478	5.622123	49.464834	0.0096315
S. araucana-E. peruviana	-11.300000	-33.221355	10.621355	0.5485567
S. lessonii-E. peruviana	-3.595652	-25.517008	18.325703	0.9873329
S. araucana-F. crassa	-38.843478	-60.764834	-16.922123	0.0002994
S. lessonii-F. crassa	-31.139130	-53.060486	-9.217775	0.0032046
S. lessonii-S. araucana	7.704348	-14.217008	29.625703	0.8283245

Table S2: Tukey post hoc test multiple comparisons of protein content of the mollusk pedal mucus. 95% family-wise confidence level.

Comparisons	diff	lwr	upr	p adj
E. peruviana-C. granosus	0.2441584	-0.9536288	1.4419455	0.9718389
F. crassa-C. granosus	1.3365920	0.1388048	2.5343791	0.0241524
S. araucana-C. granosus	-0.2691531	-1.4669403	0.9286340	0.9601363
S. lessonii-C. granosus	0.6728711	-0.5249160	1.8706583	0.4669652
F. crassa-E. peruviana	1.0924336	-0.1053536	2.2902208	0.0845680
S. araucana-E. peruviana	-0.5133115	-1.7110987	0.6844757	0.7045129
S. lessonii-E. peruviana	0.4287128	-0.7690744	1.6265000	0.8189422
S. araucana-F. crassa	-1.6057451	-2.8035323	-0.4079579	0.0054904
S. lessonii-F. crassa	-0.6637208	-1.8615080	0.5340663	0.4801524
S. lessonii-S. araucana	0.9420243	-0.2557629	2.1398114	0.1695123

Supplement 9.

Figure S1. Partition plot of the Linear Discriminant Analyses (LDA) between the Carbohydrates and Proteins content (µg ml^{-1}) of the pedal mucus of (**A**) *Chiton granosus,* (**B**) *Echinolittorina peruviana,* (**C**) *Fissurella crassa*, (**D**) *Scurria araucana,* and (**E**) *Siphonaria lessonii.* Error rate: 0.5.

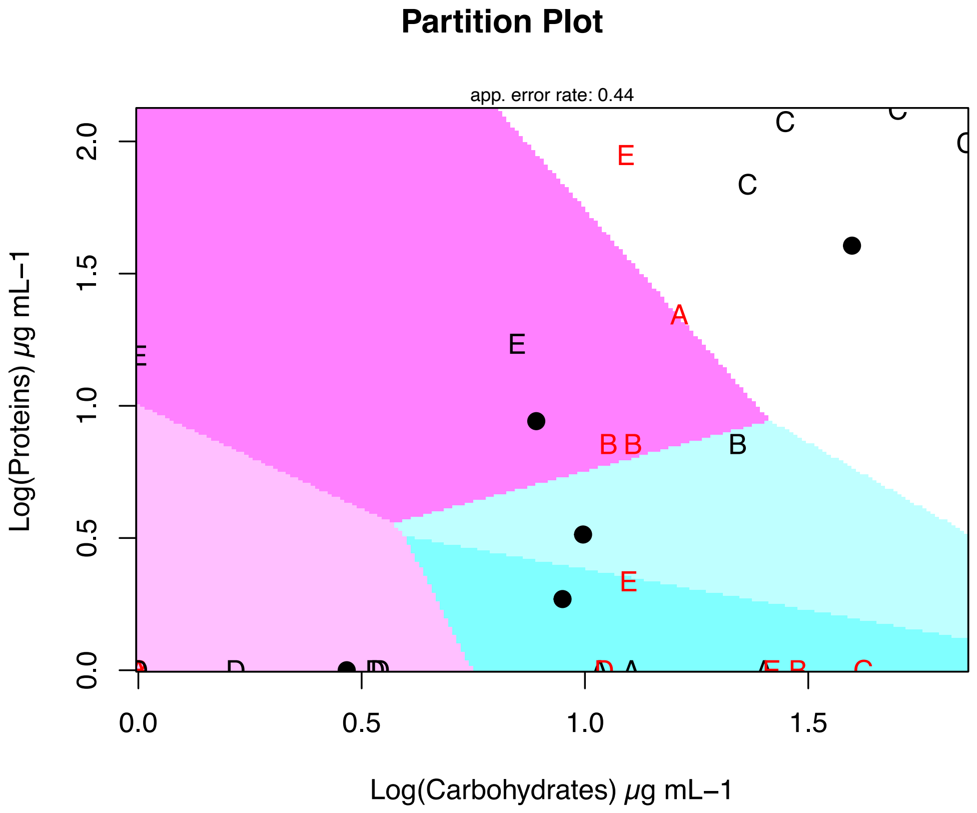

Supplement: Supplemental Information 1 [file peerj-10-13642-s001.pdf]
